# The (cost) effectiveness of procedural sedation and analgesia versus general anaesthesia for hysteroscopic myomectomy, a multicentre randomised controlled trial: PROSECCO trial, a study protocol

**DOI:** 10.1186/s12905-019-0742-1

**Published:** 2019-03-22

**Authors:** Julia F. van der Meulen, Marlies Y. Bongers, Sjors F. P. J. Coppus, Judith E. Bosmans, José M. C. Maessen, Katrien Oude Rengerink, Lucilla E. Overdijk, Celine M. Radder, Lucet F. van der Voet, Nicol A. C. Smeets, Huib A. A. M. van Vliet, Wouter J. K. Hehenkamp, Arentje P. Manger, Wilbert A. Spaans, Erica A. Bakkum, Nicole Horrée, Justine M. Briët, Jan Willem van der Steeg, Helen S. Kok

**Affiliations:** 1 Department of Obstetrics & Gynaecology, Máxima Medical Centre, PO Box 777, 5500 MB Veldhoven, The Netherlands; 2 Grow school of oncology and developmental biology, MUMC, Maastricht, The Netherlands; 3 Department of Obstetrics & Gynaecology, UMC St Radboud, Nijmegen, The Netherlands; 4 Department of Health Sciences, Section of Health Economics & Health Technology Assessment, VU Medical Centre, Amsterdam, The Netherlands; 5 Department of Quality and Safety, MUMC, Maastricht, The Netherlands; 6 Julius Center of Health Sciences and Primary Care, UMC Utrecht, Utrecht, The Netherlands; 7 Department of Obstetrics & Gynaecology, Academic Medical Centre, Amsterdam, the Netherlands; 8 Department of Anaesthesiology, Academic Medical Centre, Amsterdam, The Netherlands; 9 Department of Obstetrics & Gynaecology, Onze Lieve Vrouwe Gasthuis West, Amsterdam, The Netherlands; 10 Department of Obstetrics & Gynaecology, Deventer Ziekenhuis, Deventer, The Netherlands; 11 Department of Obstetrics & Gynaecology, Zuyderland Medical Centre, Heerlen, The Netherlands; 12 Department of Obstetrics & Gynaecology, Catharina Ziekenhuis, Eindhoven, The Netherlands; 13 Department of Obstetrics & Gynaecology, VU Medical Centre, Amsterdam, The Netherlands; 14 Department of Obstetrics & Gynaecology, Diakonessenhuis, Utrecht, The Netherlands; 15 Department of Obstetrics & Gynaecology, MUMC, Maastricht, The Netherlands; 16 Department of Obstetrics & Gynaecology, Onze Lieve Vrouwe Gasthuis Oost, Amsterdam, The Netherlands; 17 Department of Obstetrics & Gynaecology, Flevoziekenhuis, Almere, The Netherlands; 18 Department of Obstetrics & Gynaecology, Ziekenhuisgroep Twente, Almelo, The Netherlands; 19 Department of Obstetrics & Gynaecology, Jeroen Bosch Ziekenhuis, ‘s-Hertogenbosch, The Netherlands; 20 Department of Obstetrics & Gynaecology, Alrijne Ziekenhuis, Leiden, The Netherlands

**Keywords:** Submucosal fibroids, Hysteroscopic myomectomy, Procedural sedation and analgesia, General anaesthesia

## Abstract

**Background:**

In women with abnormal uterine bleeding, fibroids are a frequent finding. In case of heavy menstrual bleeding and presence of submucosal type 0–1 fibroids, hysteroscopic resection is the treatment of first choice, as removal of these fibroids is highly effective. Hysteroscopic myomectomy is currently usually performed in the operating theatre. A considerable reduction in costs and a higher patient satisfaction are expected when procedural sedation and analgesia with propofol (PSA) in an outpatient setting is applied. However, both safety and effectiveness – including the necessity for re-intervention due to incomplete resection – have not yet been evaluated.

**Methods:**

This study is a multicentre randomised controlled trial with a non-inferiority design and will be performed in the Netherlands. Women > 18 years with a maximum of 3 symptomatic type 0 or 1 submucosal fibroids with a maximum diameter of 3.5 cm are eligible to participate in the trial. After informed consent, 205 women will be randomised to either hysteroscopic myomectomy using procedural sedation and analgesia with propofol in an outpatient setting or hysteroscopic myomectomy using general anaesthesia in a clinical setting in the operating theatre.

Primary outcome will be the percentage of complete resections, based on transvaginal ultrasonography 6 weeks postoperatively. Secondary outcomes are cost effectiveness, menstrual blood loss (Pictorial blood assessment chart), quality of life, pain, return to daily activities/work, hospitalization, (post) operative complications and re-interventions. Women will be followed up to one year after hysteroscopic myomectomy.

**Discussion:**

This study may demonstrate comparable effectiveness of hysteroscopic myomectomy under procedural sedation and analgesia versus general anaesthesia in a safe and patient friendly environment, whilst achieving a significant cost reduction.

**Trial registration:**

Dutch trial register, number NTR5357. Registered 11th of August 2015.

**Electronic supplementary material:**

The online version of this article (10.1186/s12905-019-0742-1) contains supplementary material, which is available to authorized users.

## Background

In women with abnormal uterine bleeding, fibroids are a frequent finding. In case of heavy menstrual bleeding and presence of submucosal type 0–1 fibroids, hysteroscopic resection is the treatment of first choice, as removal of these fibroids is highly effective [1]. Hysteroscopic myomectomy is performed in an estimated 3000 women annually in the Netherlands. In the literature, a reduction of symptoms has been reported in 70–99% of the procedures [1–6]. The wide range of this success rate reflects the inclusion of all types of submucosal fibroids in the studies, including type 2 submucosal fibroids of which removal can be more complicated due to their extension into the myometrium. In case of submucosal fibroids hysteroscopic myomectomy is recommended as first choice treatment by the Dutch guideline on Heavy Menstrual Bleeding [7].

In the last decades, there has been a trend in moving hysteroscopic surgery from a clinical setting with general or spinal anaesthesia to an outpatient setting. Especially hysteroscopic surgery of smaller diameter polyps (< 2 cm) and low grade adhesions are eligible for resection in an outpatient setting [8–10]. Even for smaller type 0 and 1 submucosal fibroids it has been reported that these can be successfully removed in an outpatient setting under local anaesthesia [11]. However, clinical setting is still necessary for larger fibroids that generally require the use of larger diameter instruments such as a resectoscope and hence the need of cervical dilatation up to 9 mm and general or spinal anaesthesia.

Procedural sedation and analgesia (PSA) is used for a wide variety of interventional procedures in multiple settings outside the operating theatre. In gynaecology, the use of PSA has also become more popular since technical and instrumental improvements have significantly increased the feasibility and acceptability of hysteroscopy in outpatient setting [12, 13]. Until now, PSA for resection of fibroids is not commonly used.

A considerable cost reduction is expected when PSA will be applied for hysteroscopic resection of fibroids: the use of PSA means that hysteroscopic myomectomy can be moved away from the operating theatre to an outpatient setting. Hence less dedicated personnel is needed. Other potential advantages of hysteroscopic fibroid resection with PSA are avoidance of general anaesthesia and its associated risks, a shorter recovery time – resulting in a shorter hospital stay –, faster return to mobility, full fitness and normal activities. Finally, waiting lists for major surgery will be reduced by averting the need for the operating theatre for minor procedures.

Due to the abovementioned factors higher patient satisfaction is also expected, as both hospital stay and time-to-work are shorter and side effects of general anaesthesia such as nausea are reduced.

The use of PSA seems to be feasible and well-tolerated in gynaecological surgery [14–16]. However, there are no randomised controlled trials (RCTs) available on the use of PSA for hysteroscopic myomectomy. This RCT is the first trial comparing the use of PSA for hysteroscopic myomectomy with hysteroscopic myomectomy in a clinical setting under general anaesthesia.

## Methods

### Objective

The aim of this study is to compare hysteroscopic resection of symptomatic type 0 and type I submucosal fibroids under procedural sedation and analgesia with propofol in an outpatient setting with hysteroscopic myomectomy in an inpatient clinical setting using general anaesthesia. It is hypothesised that hysteroscopic myomectomy with PSA is non-inferior to the same procedure carried out under general anaesthesia. This study will also compare the cost effectiveness, pain, menstrual blood loss (pictorial blood assessment chart: PBAC score), quality of life, return to daily activities/work, hospitalization, (post) operative complications and re-interventions.

### Trial design and setting

This study is a multicentre randomised controlled trial with a non-inferiority design. A cost-effectiveness study will be performed together with the clinical study. The study is embedded in the Dutch Consortium for Healthcare Evaluation and Research in Obstetrics and Gynaecology. Participating hospitals are university and teaching hospitals in the Netherlands. Women will be randomised for hysteroscopic myomectomy either under general anaesthesia or procedural sedation and analgesia with propofol.

### Eligibility criteria

#### Inclusion criteria

The following women will be included:

- A minimum age of 18 years
- A maximum of 3 symptomatic type 0 and type 1 submucosal fibroids
- A maximum diameter of 3.5 cm (as diagnosed by transvaginal ultrasonography)
- American Society of Anaesthiologists (ASA) class 1 or 2
- Sufficient knowledge of Dutch or English language to fully understand the study and complete the questionnaires

#### Exclusion criteria

- Presence of clotting disorders
- Severe anemia (Hb under 5.0 mmol/l)
- ASA class 3 or 4

### Interventions

According to guidelines from the Health Care Inspectorate (IGZ) and Dutch Institute for Healthcare Improvement (CBO) Non-Anaesthesiologist Administered Propofol (NAAP) sedation is given and monitored by a qualified sedation practitioner [17, 18]. Non-invasive blood pressure, electrocardiogram and oxygen saturation are measured before vascular access is obtained. Propofol combined with short acting intravenous analgesia is used for procedural sedation.

An experienced surgeon performs the hysteroscopic resection by standard procedure with the use of a resectoscope or morcellation device in an office based setting. Patients are observed after the procedure by qualified personnel and discharged as soon as all the discharge criteria are met, normally within 1 to 1.5 h.

General anaesthesia can be volatile based or total intravenously, with the use of a laryngeal mask or tube. Postoperatively, patients will be observed in the recovery room and discharged home from clinic when all the discharge criteria are met. Hysteroscopic myomectomy is performed by standard procedure.

### Outcome measures

#### Primary outcome measures

Primary outcome will be the percentage of complete resections, evaluated by transvaginal ultrasonography (TVU) (contrast sonography if TVU is inconclusive) 6 weeks postoperatively. The ultrasonography will be performed by an independent gynaecologist or ultrasonographer blinded for the treatment arm and judgment of completeness by the surgeon who performed the hysteroscopic myomectomy. This TVU should be conducted 6 weeks postoperatively. A complete resection means that there are no signs of an intracavitary remaining of the fibroid (s) resected during hysteroscopic myomectomy. This follow-up visit and TVU is part of the current usual care, so no extra ultrasonography is needed for study purposes.

#### Secondary outcome measures

Secondary outcomes are cost effectiveness, pain, menstrual blood loss (PBAC score), quality of life, return to daily activities/work, hospitalization, (post) operative complications, re-interventions. These secondary parameters will be assessed by several questionnaires.

### Patient recruitment

The gynaecologist participating in the network will inform women about the study and refer the eligible women to dedicated research nurses if available. These nurses or the local investigator will counsel women, ask for written informed consent and perform randomisation to either PSA or general anaesthesia. The investigators will also organize the required treatments, depending on the result of the randomisation. Subjects can leave the study at any time for any reason if they wish to do so without any consequences. The investigator can decide to withdraw a subject from the study for urgent medical reasons.

### Randomisation

Randomisation will be performed web based with the use of a block design, with a variable block size.

### Blinding

The study will not be double-blinded, as it is impossible to blind the health care workers and women involved for the strategy to which the woman is allocated. The person performing the transvaginal ultrasonography at 6 weeks follow up, however, will be blinded for the study arm and judgement of completeness by the surgeon during the procedure.

### Data collection

Women will be asked to complete questionnaires online, through a link they receive by e-mail (it is possible to complete the questionnaires on paper when women prefer to do so).

A website dedicated to studies in the Dutch Consortium for Healthcare Evaluation and Research in Obstetrics and Gynaecology will be used for data collection. Research nurses in each of the participating centres will perform the data collection. A computer generated numeric code – to which the key is only available to the local investigator or local research nurse–will be attributed to each participant, enabling anonymous data management.

In accordance with the guidelines of the Dutch Federation of University Medical Centers (NFU) the data will be preserved for 15 years.

**Fig. 1:**
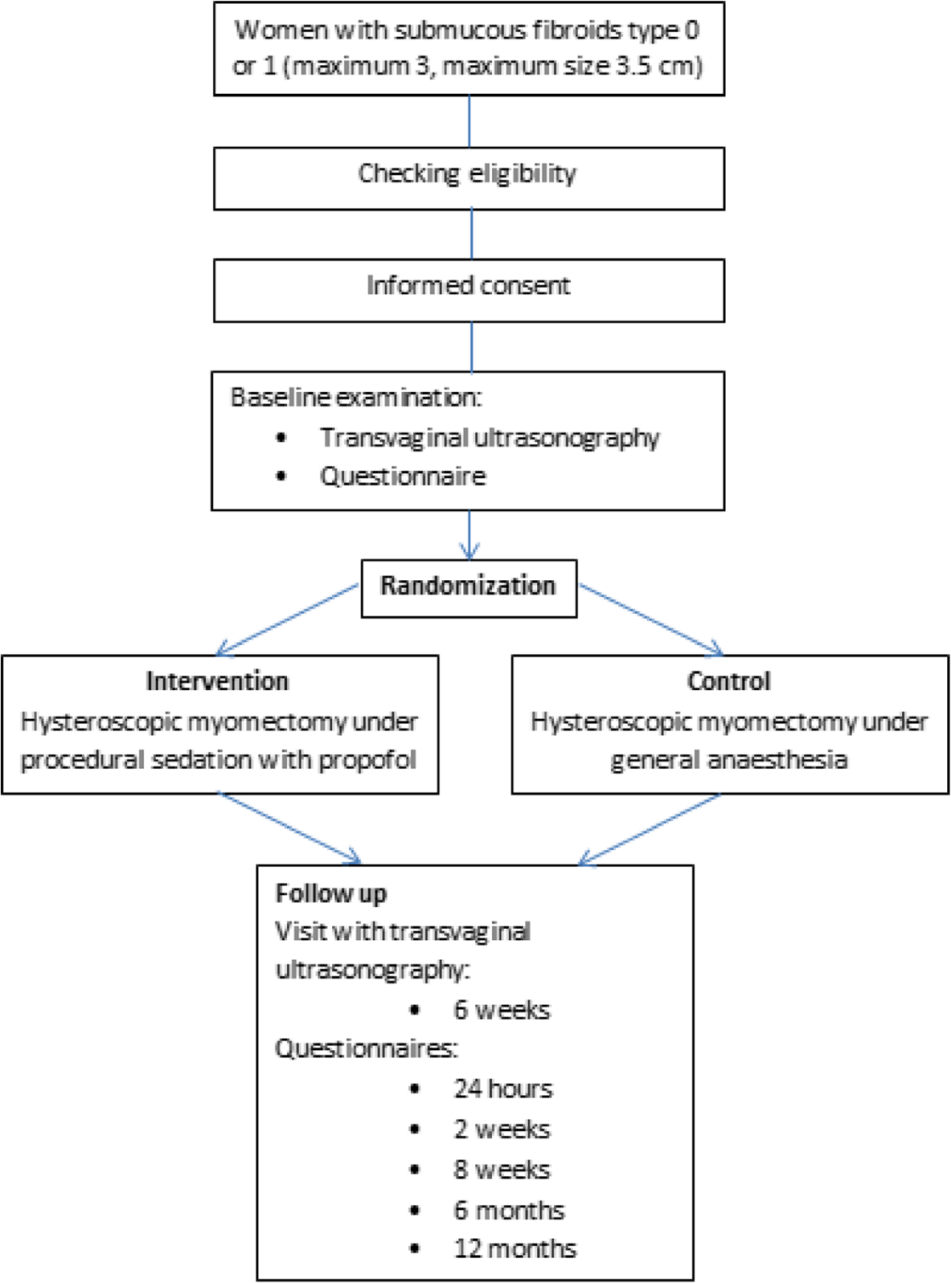
Flowchart of study design

Women will be followed from baseline (pre-operatively) up to 1 year after hysteroscopic myomectomy (Fig. 1). During follow up the following data will be collected and registered in a Case Record Form (CRF):

- Baseline characteristics

At baseline, before treatment, the following characteristics are registered: body mass index, medical history, demographics, parity, age, type and number of fibroids.

- Characteristics registered during and after the procedure

Completeness of resection as judged by surgeon, surgical complications, anaesthesiologic complications (desaturation, airway obstruction, dysrhythmias, blood pressure drops, ECG alterations), operating time, intravasation/fluid deficit, recovery time (time from end of procedure until completely awake and communicative), use of pain medication, pain intensity measured by NRS scores, duration of hospitalization (hours).

- Questionnaires

Participating women will fill in questionnaires at baseline, which will be given before the surgical procedure. During follow-up, women will fill in questionnaires at 24 h, 2 weeks, 8 weeks, 6 and 12 months after randomisation (Table 1). Questionnaires will contain:

- Short questionnaire on side effects 24 h after surgery. See Additional files 1 and 2 for an example of the Dutch and English version of this questionnaire.
- The EuroQoL (EQ-5D-5 L) questionnaire (15 questions) to assess the quality of life [19].
- Pictorial Blood Assessment Chart (PBAC) scores to assess the amount of menstrual blood loss. See Additional files 3 and 4 for an example of the Dutch and English version of this questionnaire.
- Uterine Fibroid Symptoms – Quality of Life (UFS-QoL) questionnaire (37 questions) to assess the quality of life focussing on women suffering from uterine fibroids [20]. See Additional file 5 for an example of the Dutch version of this questionnaire.
- Recovery Index (RI-10) questionnaire (10 questions): to assess a patients’ recovery process [21].
- Medical Consumption Questionnaire (iMCQ): for cost effectiveness analysis [22].
- Productivity Cost Questionnaire (iPCQ) to assess productivity loss [23].
- Short questionnaire on recurrence and re-interventions at 12 months after surgery. See Additional files 6 and 7 for an example of the Dutch and English version of this questionnaire.

**Table 1:** Questionnaires and exams at baseline and during follow up

	Side effects, NRS	EQ-5D-5 L	PBAC	UFS-QoL	RI-10	iMCQ	iPCQ	Re-intervention	TVU
Baseline		X	X	X					X
24 h	X	X			X				
2 weeks		X			X				
6 weeks									X
8 weeks		X	X	X	X	X	X		
6 months		X				X	X		
12 months		X	X			X	X	X	

### Monitoring

An independent Data Safety Monitoring Board (DSMB) will be asked to monitor the progress of the study and the safety of its participants. Accumulating data on serious adverse events will be sent to the DSMB each time data of serious adverse effects in 20 women has been received. In addition, an overview of reported SAEs will be send to the DSMB after each 50 women included and operated during the study. The DSMB will meet as required to review any expected adverse events and may ask to review outcomes or other data that may have an impact on the trial.

No formal interim analysis or efficacy will be done.

### Statistical analysis

#### Sample size

The study is designed as a non-inferiority study, in which we aim to investigate if hysteroscopic myomectomy under procedural sedation with propofol is non-inferior to the same surgical procedure under general anaesthesia. With 205 women randomized we have 90% power to demonstrate non-inferiority, based on an estimated 2.5% incomplete resections in both groups, with an non-inferiority upper limit of 10% incomplete resections defined as non-inferior (i.e. a delta of 7.5%), an alpha of 0.025 and a drop-out rate of 10%.

#### Data analysis

Data will be analysed according to the intention-to-treat principle. We will also perform a per protocol analysis, given the non-inferiority design of the study, where crossover between the groups will increase the chances of concluding non-inferiority, if in reality the treatment is not inferior. We will present the percentage of complete resections at 6 weeks in both groups, with according relative risks and 95% confidence interval. Differences will be tested with the Chi-square test, or, if the expected cell count is low using the Fisher exact test. We will also calculate the relative risk, adjusted for the resection technique which was used. Stratification will be performed by resection technique (resection or morcellation). Complications during and after surgery, and re-interventions will be categorized. The Data and Safety Monitoring Board will evaluate serious adverse events.

Pain intensity after the procedure and at discharge will be reported as means with SD, risk differences between both groups will be calculated with according 95% confidence interval. Time to recovery and pain will be visualized in a graph, and analyzed using a mixed model, that can take into account repeated measures in the same woman over time. The quality of life and PBAC scores will be analyzed according the developed algorithms. We will perform a subgroup analysis for fibroid size (< 2 cm versus ≥2 cm) and for parity (nulliparous versus multiparous women).

### Economic evaluation

This economic evaluation aims to link the difference in societal and healthcare costs to the difference in clinical effects between PSA and general anaesthesia. A cost-effectiveness and cost-utility analysis will be carried out with a time horizon of 1 year. Thus, discounting is not necessary. Costs will be measured from a societal perspective using internet questionnaires based on the iMCQ after 8 weeks, 6 and 12 months of follow-up. Direct costs consist of costs of primary and hospital care as well as costs of complementary care and home care. Indirect costs involve costs caused by being absent from paid and unpaid work, or by presenteeism. Estimation of indirect costs will be done by using the friction cost approach. The standard prices presented in the Dutch costing guidelines [24] will be used to value health care utilization and prices of the Royal Dutch Society for Pharmacy [25] will be used to value medication use.

Societal costs will be related to the following effect measures in the economic evaluation: percentage of complete resections and quality-adjusted life-years (QALYs) based on the Dutch tariff for the EuroQol (EQ-5D-5 L) [26, 27]. The analysis will be done according to the intention-to-treat principle. Missing cost and effect data will be imputed using multiple imputation. The difference in mean total costs between the two groups will be divided by the difference in mean effects in order to calculate the Incremental cost-effectiveness ratios (ICERs). Estimation of 95% confidence intervals around cost differences will be done by bootstrapping with 5000 replications. This will also be used to estimate the uncertainty around the ICERs, which will be demonstrated on cost-effectiveness planes. Estimation of cost-effectiveness acceptability curves will be performed to present the likelihood that the intervention is cost-effective compared to the current standard care for a spectrum of different ceiling ratios [28]. .Adjustment for confounders and effect modifiers will be done if necessary.

## Discussion

Fibroids are a frequent finding in women with abnormal uterine bleeding. The Dutch Guideline ‘Heavy menstrual bleeding’ recommends hysteroscopic myomectomy as treatment of first choice in case of symptomatic submucous fibroids [7]. In the current situation, women are usually admitted into daycare and they are operated under general anaesthesia. This requires an operating theatre with a full anaesthetic team. Procedural sedation has never been evaluated for hysteroscopic myomectomy regarding both effectiveness (complete resection) and cost-effectiveness. The Dutch guideline committee suggests that local anaesthesia or ‘conscious sedation’ can be taken into consideration for hysteroscopic myomectomy based on indirect evidence from other areas in healthcare [7]. This non-inferiority RCT is the first trial about hysteroscopic myomectomy for symptomatic type 0 or I submucosal fibroids in an outpatient setting using procedural sedation with propofol compared to the current standard hysteroscopic myomectomy in daycare using general anaesthesia. Several advantages are expected when performing hysteroscopic myomectomy under PSA compared to general anaesthesia. Due to this study design and outcome measures, the results will be applicable for a large group of women suffering from abnormal uterine bleeding caused by submucosal fibroids.

## Additional files

Additional file 1: Questionnaire on side effects 24 h after surgery. (PDF 129 kb)

Additional file 2: Questionnaire on side effects 24 h after surgery. (PDF 109 kb)

Additional file 3: Pictorial Blood Assessment Chart (PBAC). (PDF 134 kb)

Additional file 4: Pictorial Blood Assessment Chart (PBAC). (PDF 78 kb)

Additional file 5: Uterine Fibroid Symptoms – Quality of Life (UFS-QoL) questionnaire. (PDF 265 kb)

Additional file 6: Questionnaire on recurrence and re-interventions 12 months after surgery. (PDF 76 kb)

Additional file 7: Questionnaire on recurrence and re-interventions 12 months after surgery. (PDF 78 kb)

## Abbreviations

ASA: American Society of Anaesthesiologists
iMCQ: Medical Consumption Questionnaire
iPCQ: Productivity Cost Questionnaire
NAAP: Non-Anaesthesiologist Administered Propofol
PBAC: Pictorial Blood Assessment Chart
PSA: Procedural sedation and analgesia
RCT: Randomised controlled trial
TVU: Transvaginal ultrasonography
UFS-QoL: Uterine fibroid symptoms – quality of life questionnaire
WMO: Medical Research Involving Human Subjects Act (in Dutch: Wet Medisch-wetenschappelijk Onderzoek met Mensen)
