# Supplementary material for: The (cost) effectiveness of procedural sedation and analgesia versus general anaesthesia for hysteroscopic myomectomy, a multicentre randomised controlled trial: PROSECCO trial, a study protocol
Source: BMC Womens Health. 2019 Mar 22;19:46. doi: 10.1186/s12905-019-0742-1 (PMC6431064; doi:10.1186/s12905-019-0742-1)
Supplement: Supplementary file 1 — Questionnaire on side effects 24 h after surgery. (PDF 129 kb) [file 12905_2019_742_MOESM1_ESM.pdf]

máxima medisch centrum

Vragenlijst bijwerkingen operatie na 24 uur.

1. Datum van invullen: ....(dd/mm/jaar)

2. Hoe misselijk bent u op dit moment?
Omcirkel hieronder alstublieft het getal dat het beste uw mate van misselijkheid op dit moment aangeeft. Een ‘0’ betekent niet misselijk, een ‘10’ betekent de meest denkbare misselijkheid.

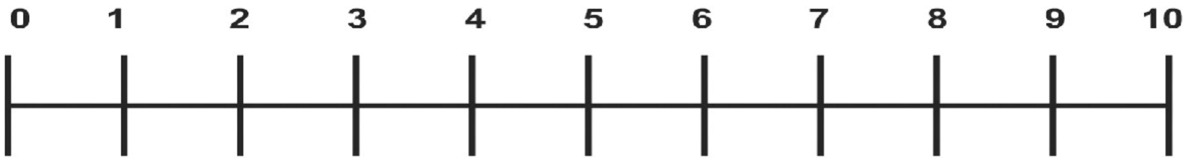

Niet misselijk

Meest denkbare misselijkheid

3. Hebt u moeten overgeven na de operatie?

Ja ☐

Nee ☐

Indien u de vorige vraag met ja hebt beantwoord:

4. Hoe vaak hebt u na de operatie moeten overgeven? ………………….. keer

5. Hoeveel pijn hebt u op dit moment?
Omcirkel hieronder alstublieft het getal dat het beste uw pijn op dit moment aangeeft. Een ‘0’ betekent geen pijn, een ‘10’ betekent de meest denkbare pijn.

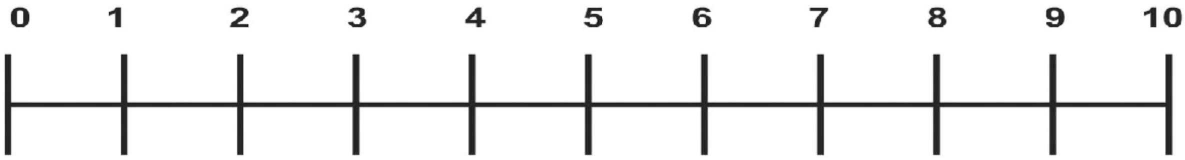

Geen pijn

Meest denkbare pijn
