# Supplementary material for: The (cost) effectiveness of procedural sedation and analgesia versus general anaesthesia for hysteroscopic myomectomy, a multicentre randomised controlled trial: PROSECCO trial, a study protocol
Source: BMC Womens Health. 2019 Mar 22;19:46. doi: 10.1186/s12905-019-0742-1 (PMC6431064; doi:10.1186/s12905-019-0742-1)
Supplement: Supplementary file 2 — Questionnaire on side effects 24 h after surgery. (PDF 109 kb) [file 12905_2019_742_MOESM2_ESM.pdf]

PROSECCO

Questionnaire on side effects 24 hours after hysteroscopic myomectomy

1. Date while filling in this questionnaire: ....(day/month/year)

2. How nauseous are you at the moment?

At this scale, please mark the number that indicates how nauseous you feel at the moment. ‘0’ means you don’t experience any nausea, ’10’ means the most extreme nausea you can imagine.

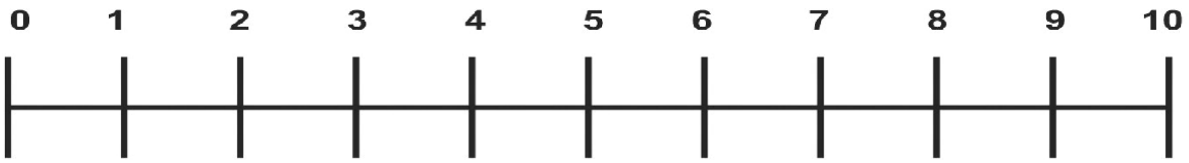

No nausea

Most extreme nausea

3. Did you have to throw up after the surgery?
   - Yes ☐
   - No ☐

Did you check yes? Go to question 4. Otherwise skip to question 5.

4. How many times did you have to throw up after the surgery?…………………..times

5. How much pain do you experience at the moment?

At this scale, please mark the number that indicates how much pain you experience at the moment. ‘0’ means you don’t experience any pain, ’10’ means the most extreme pain you can imagine.

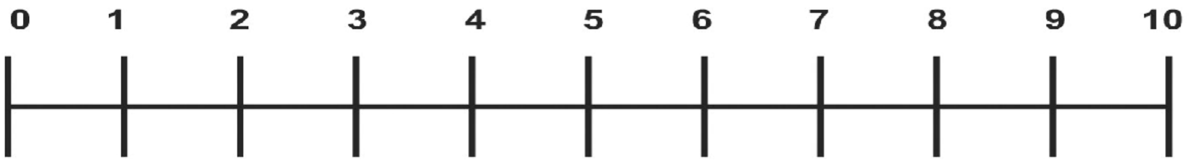

No pain

Worst pain you can imagine
