# Supplementary material for: The (cost) effectiveness of procedural sedation and analgesia versus general anaesthesia for hysteroscopic myomectomy, a multicentre randomised controlled trial: PROSECCO trial, a study protocol
Source: BMC Womens Health. 2019 Mar 22;19:46. doi: 10.1186/s12905-019-0742-1 (PMC6431064; doi:10.1186/s12905-019-0742-1)
Supplement: Supplementary file 3 — Pictorial Blood Assessment Chart (PBAC). (PDF 134 kb) [file 12905_2019_742_MOESM3_ESM.pdf]

MENSTRUATIESCOREKAART MAAND______JAAR_____PT. INITIALEN______ GEBOORTEDATUM __-__-____ Case number: __ - ____

Handleiding voor het gebruik van de menstruatiescorekaart **Follow-up: 3 / 6 / 12 maanden** *(omcirkelen wat van toepassing is)*

- U kunt met deze kaart de menstruatie gedurende één maand bijhouden.
- Gebruik bij voorkeur maxi maandverband en/of maxi tampons.
- Wees niet zuinig met het gebruik van verbanden en tampons en probeer hierin zoveel mogelijk al het menstruatiebloed op te vangen.
- Denk eraan ook ‘s nachts voldoende verband/tampons te gebruiken, zo nodig beide tegelijk.
- Houd bij voorkeur een tampon in tijdens plassen, ontlasting, douche of bad en verwissel pas daarna.
- Elke keer wanneer u een verband of tampon verwijdert, vergelijkt u het zichtbare bloedverlies met de afbeeldingen op de menstruatiescorekaart en zet u een verticaal streepje (‘turven’) in het hokje naast het plaatje dat het meest daarmee overeenkomt. De cijfers in de bovenste rij komen overeen met de dag van de maand.
- Op de dagen dat u alleen wat licht bloedverlies heeft (spotting) kunt u een kruisje zetten in het vakje van die dag.

☐ **Geen bloeding deze maand** **OF** **BLOEDING OP BEPAALDE DAGEN VAN DEZE MAAND INVULLEN:**

**VERBAND**	**1**	**2**	**3**	**4**	**5**	**6**	**7**	**8**	**9**	**10**	**11**	**12**	**13**	**14**	**15**	**16**	**17**	**18**	**19**	**20**	**21**	**22**	**23**	**24**	**25**	**26**	**27**	**28**	**29**	**30**	**31**
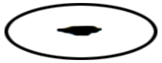																															
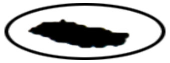																															
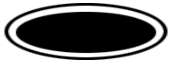																															

**TAMPON**	**1**	**2**	**3**	**4**	**5**	**6**	**7**	**8**	**9**	**10**	**11**	**12**	**13**	**14**	**15**	**16**	**17**	**18**	**19**	**20**	**21**	**22**	**23**	**24**	**25**	**26**	**27**	**28**	**29**	**30**	**31**
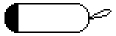																															
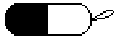																															
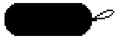																															

STOLSELS (x = Ja)																															
SPOTTING (x = Ja)																															
PIJNSCORE*																															

* Pijnscore: 0 = geen, 1 = mild, 2 = middelmatig, 3 = ernstig
