# Supplementary material for: The (cost) effectiveness of procedural sedation and analgesia versus general anaesthesia for hysteroscopic myomectomy, a multicentre randomised controlled trial: PROSECCO trial, a study protocol
Source: BMC Womens Health. 2019 Mar 22;19:46. doi: 10.1186/s12905-019-0742-1 (PMC6431064; doi:10.1186/s12905-019-0742-1)
Supplement: Supplementary file 4 — Pictorial Blood Assessment Chart (PBAC). (PDF 78 kb) [file 12905_2019_742_MOESM4_ESM.pdf]

Pictorial Blood Assessment Chart PROSECCO MONTH_______ YEAR _______ Patient ID ___________________________

How to use the Pictorial Blood Assessment Chart:

- This chart enables you to record your during your period in one month.
- Preferably use maxi sanitary towels and/or tampons.
- Please try to use a sufficient amount of sanitary towels and/or tampons and try to collect most of the blood you lose during your period in these.
- Please be reminded to use a sufficient amount of sanitary towels/tampons during the night as well, if necessary both at the same time.
- Preferably leave in your tampon during peeing, defecation, taking a shower or bath and change afterwards.
- During the course of your period record your use of tampons and sanitary towels by placing a tally mark under the day next to the box that represents how stained your sanitary materials are each time you change them. The numbers in the upper row represent the day of the month.
- On days you experience very little blood loss (spotting) only, you can place a mark in the box that represents that day.

SANITARY TOWEL	1	2	3	4	5	6	7	8	9	10	11	12	13	14	15	16	17	18	19	20	21	22	23	24	25	26	27	28	29	30	31
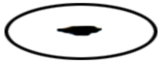																															
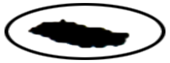																															
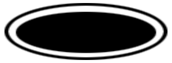																															

TAMPON	1	2	3	4	5	6	7	8	9	10	11	12	13	14	15	16	17	18	19	20	21	22	23	24	25	26	27	28	29	30	31
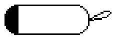																															
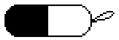																															
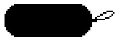																															

CLOTS (x = yes)																															
SPOTTING (x = yes)																															
PAINSCORE*																															

* Painscore: 0 = none, 1 = little, 2 = moderate, 3 = severe
