# Supplementary material for: The (cost) effectiveness of procedural sedation and analgesia versus general anaesthesia for hysteroscopic myomectomy, a multicentre randomised controlled trial: PROSECCO trial, a study protocol
Source: BMC Womens Health. 2019 Mar 22;19:46. doi: 10.1186/s12905-019-0742-1 (PMC6431064; doi:10.1186/s12905-019-0742-1)
Supplement: Supplementary file 5 — Uterine Fibroid Symptoms – Quality of Life (UFS-QoL) questionnaire. (PDF 265 kb) [file 12905_2019_742_MOESM5_ESM.pdf]

Initialen patiënt: __________ **Patiëntnummer:** __________

Datum: __________

VRAGENLIJST OVER SYMPTOMEN EN GEZONDHEIDSGERELATEERDE KWALITEIT VAN LEVEN BIJ MYOMEN (UFS-QOL NL)

Hieronder worden symptomen beschreven die vrouwen met myomen ervaren. Beoordeel elk symptoom aan de hand van uw myomen of menstruatiecyclus. Het is de bedoeling dat u steeds aangeeft hoeveel last u de afgelopen drie maanden van het betreffende symptoom heeft gehad.

Er zijn geen goede of foute antwoorden. Beantwoord elke vraag door het keuzevakje aan te vinken (✓) dat het beste bij uw antwoord past. Als een vraag niet op u van toepassing is, vinkt u het antwoord "Niet van toepassing" aan.

	**Hoeveel last heeft u de afgelopen drie maanden gehad van…**	**Geen**	**Een klein beetje**	**Enigszins**	**Veel**	**Heel veel**	**Niet van toepassing**
1.	Hevig bloedverlies tijdens uw menstruatieperiode?	☐ 1	☐ 2	☐ 3	☐ 4	☐ 5	☐ n.v.t.
2.	Verlies van bloedstolsels tijdens uw menstruatieperiode?	☐ 1	☐ 2	☐ 3	☐ 4	☐ 5	☐ n.v.t.
3.	Schommelingen in de duur van uw menstruatieperiode in vergelijking met uw vorige cyclus?	☐ 1	☐ 2	☐ 3	☐ 4	☐ 5	☐ n.v.t.
4.	Schommelingen in de tijd tussen twee menstruatieperiodes in vergelijking met vorige cycli?	☐ 1	☐ 2	☐ 3	☐ 4	☐ 5	☐ n.v.t.
5.	Een gespannen of drukkend gevoel in het bekkengebied?	☐ 1	☐ 2	☐ 3	☐ 4	☐ 5	☐ n.v.t.
6.	Veelvuldig plassen overdag?	☐ 1	☐ 2	☐ 3	☐ 4	☐ 5	☐ n.v.t.
7.	Veelvuldig plassen 's nachts?	☐ 1	☐ 2	☐ 3	☐ 4	☐ 5	☐ n.v.t.
8.	Een gevoel van vermoeidheid?	☐ 1	☐ 2	☐ 3	☐ 4	☐ 5	☐ n.v.t.

De volgende vragen gaan over de invloed die symptomen van myomen hebben op uw leven. Geef bij elke vraag aan welk antwoord het beste past bij uw ervaringen met myomen in de afgelopen drie maanden.

Er zijn geen goede of foute antwoorden. Beantwoord elke vraag door het keuzevakje aan te vinken (✓) dat het beste bij uw antwoord past. Als een vraag niet op u van toepassing is, vinkt u het antwoord "Niet van toepassing" aan.

**Gedurende de afgelopen drie maanden, hoe vaak hebben uw symptomen, gerelateerd aan myomen…**		**Nooit**	**Heel af en toe**	**Soms**	**Vaak**	**Altijd**	**Niet van toepassing**
9.	U onzeker gemaakt over het beginmoment en de duur van uw menstruatieperiode?	☐ 1	☐ 2	☐ 3	☐ 4	☐ 5	☐ n.v.t.
10.	U onzeker gemaakt over het maken van reizen?	☐ 1	☐ 2	☐ 3	☐ 4	☐ 5	☐ n.v.t.
11.	Uw lichamelijke activiteiten belemmerd?	☐ 1	☐ 2	☐ 3	☐ 4	☐ 5	☐ n.v.t.
12.	Geleid tot vermoeidheid of uitputting?	☐ 1	☐ 2	☐ 3	☐ 4	☐ 5	☐ n.v.t.
13.	Gemaakt dat u de tijd die u normaal gesproken besteedt aan sport of andere vormen van lichaamsbeweging, verminderde?	☐ 1	☐ 2	☐ 3	☐ 4	☐ 5	☐ n.v.t.
14.	U het gevoel gegeven dat u uw leven niet zelf in de hand heeft?	☐ 1	☐ 2	☐ 3	☐ 4	☐ 5	☐ n.v.t.
15.	U bezorgd gemaakt over het vies worden van uw ondergoed?	☐ 1	☐ 2	☐ 3	☐ 4	☐ 5	☐ n.v.t.
16.	U het gevoel gegeven dat u minder productief bent?	☐ 1	☐ 2	☐ 3	☐ 4	☐ 5	☐ n.v.t.
17.	Geleid tot een gevoel van sufheid of slaperigheid overdag?	☐ 1	☐ 2	☐ 3	☐ 4	☐ 5	☐ n.v.t.
18.	U het gevoel gegeven dat u bent aangekomen?	☐ 1	☐ 2	☐ 3	☐ 4	☐ 5	☐ n.v.t.
19.	U het gevoel gegeven dat het moeilijk is om uw dagelijkse bezigheden te verrichten?	☐ 1	☐ 2	☐ 3	☐ 4	☐ 5	☐ n.v.t.
20.	Uw sociale activiteiten belemmerd?	☐ 1	☐ 2	☐ 3	☐ 4	☐ 5	☐ n.v.t.
21.	U het gevoel gegeven dat uw buik groter is dan normaal en er anders uitziet?	☐ 1	☐ 2	☐ 3	☐ 4	☐ 5	☐ n.v.t.
22.	U bezorgd gemaakt over het vies worden van beddengoed?	☐ 1	☐ 2	☐ 3	☐ 4	☐ 5	☐ n.v.t.

Gedurende de afgelopen drie maanden, hoe vaak hebben uw symptomen, gerelateerd aan myomen…		Nooit	Heel af en toe	Soms	Vaak	Altijd	Niet van toepassing
23.	U verdrietig, moedeloos of wanhopig gemaakt?	☐ 1	☐ 2	☐ 3	☐ 4	☐ 5	☐ n.v.t.
24.	U somber gemaakt?	☐ 1	☐ 2	☐ 3	☐ 4	☐ 5	☐ n.v.t.
25.	U uitgeput?	☐ 1	☐ 2	☐ 3	☐ 4	☐ 5	☐ n.v.t.
26.	Geleid tot bezorgdheid of ongerustheid over uw gezondheid?	☐ 1	☐ 2	☐ 3	☐ 4	☐ 5	☐ n.v.t.
27.	Geleid tot het zorgvuldiger plannen van uw activiteiten?	☐ 1	☐ 2	☐ 3	☐ 4	☐ 5	☐ n.v.t.
28.	U een gevoel van ongemak gegeven omdat u altijd extra maandverband, tampons of ondergoed mee moet nemen om ongelukjes te voorkomen?	☐ 1	☐ 2	☐ 3	☐ 4	☐ 5	☐ n.v.t.
29.	Geleid tot schaamte?	☐ 1	☐ 2	☐ 3	☐ 4	☐ 5	☐ n.v.t.
30.	U onzeker gemaakt over uw toekomst?	☐ 1	☐ 2	☐ 3	☐ 4	☐ 5	☐ n.v.t.
31.	U prikkelbaar gemaakt?	☐ 1	☐ 2	☐ 3	☐ 4	☐ 5	☐ n.v.t.
32.	U bezorgd gemaakt over het vies worden van uw broek?	☐ 1	☐ 2	☐ 3	☐ 4	☐ 5	☐ n.v.t.
33.	Invloed gehad op de maat van de kleding die u tijdens uw menstruatieperiode draagt?	☐ 1	☐ 2	☐ 3	☐ 4	☐ 5	☐ n.v.t.
34.	U het gevoel gegeven dat u uw gezondheid niet zelf in de hand heeft?	☐ 1	☐ 2	☐ 3	☐ 4	☐ 5	☐ n.v.t.
35.	U een gevoel van zwakte gegeven, een totaal gebrek aan energie?	☐ 1	☐ 2	☐ 3	☐ 4	☐ 5	☐ n.v.t.
36.	Uw zin in seks verminderd?	☐ 1	☐ 2	☐ 3	☐ 4	☐ 5	☐ n.v.t.
37.	Geleid tot het vermijden van seksuele contacten?	☐ 1	☐ 2	☐ 3	☐ 4	☐ 5	☐ n.v.t.
