# Supplementary material for: The (cost) effectiveness of procedural sedation and analgesia versus general anaesthesia for hysteroscopic myomectomy, a multicentre randomised controlled trial: PROSECCO trial, a study protocol
Source: BMC Womens Health. 2019 Mar 22;19:46. doi: 10.1186/s12905-019-0742-1 (PMC6431064; doi:10.1186/s12905-019-0742-1)
Supplement: Supplementary file 6 — Questionnaire on recurrence and re-interventions 12 months after surgery. (PDF 76 kb) [file 12905_2019_742_MOESM6_ESM.pdf]

PROSECCO

Vragenlijst evaluatie recidief na hysteroscopische myoomresectie

Het is nu een jaar geleden dat u de hysteroscopische myoomresectie heeft ondergaan (het verwijderen van de vleesboom met een kijkbuisje via de vagina). Wij zijn benieuwd of deze vleesboom het afgelopen jaar is teruggekomen en of u hiervoor een behandeling hebt ondergaan. Geeft u daarom zo nauwkeurig mogelijk antwoord op onderstaande vragen, door het juiste hokje aan te vinken.

1. Datum van invullen: ....(dd/mm/jaar)

2. Is de vleesboom waarvoor u behandeld bent het afgelopen jaar teruggekomen?

   Ja ☐

   Nee ☐

Indien u de vorige vraag met ja hebt beantwoord, ga dan verder met vraag 3.
Indien u de vorige vraag met nee hebt behandeld, dan is dit het einde van de vragenlijst.

3. Kreeg u opnieuw klachten door het terugkomen van de vleesboom?

   Ja ☐

   Nee ☐

Indien u de vorige vraag met ja hebt beantwoord:

4. Hebt u hiervoor een behandeling ondergaan?

   Ja ☐

   Nee ☐

Indien u de vorige vraag met ja hebt beantwoord:

5. Welke behandeling hebt u ondergaan?

   ☐ Gestart met medicatie vanwege uw menstruatiecyclus? (de pil, MIRENA spiraaltje, cyklokapron)

   Datum: …………..……………..

□ Het verwijderen van de vleesboom met een kijkbuisje via de vagina (hysteroscopische myoomresectie)

Datum: …………..……………..

□ Het wegbranden van het baarmoederslijmvlies (endometrium ablatie)

Datum: …………..……………..

□ Het verwijderen van de baarmoeder
- □ via de vagina
- □ via de buik
- □ met kijkbuisjes (laparoscopisch)

Datum: …………..……………..

Einde vragenlijst.
