# Supplementary material for: The (cost) effectiveness of procedural sedation and analgesia versus general anaesthesia for hysteroscopic myomectomy, a multicentre randomised controlled trial: PROSECCO trial, a study protocol
Source: BMC Womens Health. 2019 Mar 22;19:46. doi: 10.1186/s12905-019-0742-1 (PMC6431064; doi:10.1186/s12905-019-0742-1)
Supplement: Supplementary file 7 — Questionnaire on recurrence and re-interventions 12 months after surgery. (PDF 78 kb) [file 12905_2019_742_MOESM7_ESM.pdf]

PROSECCO

Questionnaire for evaluation of recurrence after hysteroscopic myomectomy

It has been a year since you underwent the hysteroscopic myomectomy (the removal of a fibroid with a small instrument through the vagina).
We would like to know if you experienced a recurrence of your fibroid and whether or not you underwent treatment for this during the last year.
To evaluate this, please answer the following questions by checking the appropriate box.

1. Date while filling in this questionnaire: ....(day/month/year)

2. Did the fibroid, that you were operated for, return in the last year?
   Yes □
   No □

Did you check yes? Go to question 3. Otherwise, this is the end of the questionnaire.

3. Did you experience new complaints because the fibroid did return?
   Yes □
   No □

Did you check yes? Go to question 4. Otherwise, this is the end of the questionnaire.

4. Did you receive treatment for this?
   Yes □
   No □

Did you check yes? Go to question 5. Otherwise, this is the end of the questionnaire.

5. Which treatment did you receive?

   □ Start with medication because of your menstrual cycle? (oral contraception, MIRENA iud, cyklokapron)

   Date: …………..……………..

   □ Removal of the fibroid with a small instrument through the vagina (hysteroscopic myomectomy)

   Date: …………..……………..

   □ Operation to remove or thin the endometrium (the lining of the uterus (womb): endometrial ablation

   Date: …………..……………..

   □ Removal of the uterus (womb)

□ through the vagina: vaginal hysterectomy
□ though a cut in your tommy: abdominal hysterectomy
□ by keyhole surgery: laparoscopic hysterectomy

Date: …………..……………..

End of the questionnaire
